# The impact of primary percutaneous coronary intervention strategies during ST-elevation myocardial infarction on the prevalence of coronary microvascular dysfunction

**DOI:** 10.1038/s41598-023-47343-x

**Published:** 2023-11-16

**Authors:** Ali Aldujeli, Ayman Haq, Tsung-Ying Tsai, Ingrida Grabauskyte, Vacis Tatarunas, Kasparas Briedis, Sumit Rana, Ramunas Unikas, Anas Hamadeh, Patrick W. Serruys, Emmanouil S. Brilakis

**Affiliations:** 1 Lithuanian University of Health Sciences, Sukileliu pr. 15, 50161 Kaunas, Lithuania; 2 Abbott Northwestern Hospital/Minneapolis Heart Institute Foundation, Minneapolis, MN USA; 3 University of Galway, Galway, Ireland; 4 Thorndale Medical Clinic, Dublin, Ireland; 5 Heart and Vascular Specialists of North Texas, Arlington, TX USA

**Keywords:** Cardiology, Interventional cardiology

## Abstract

Coronary microvascular dysfunction (CMD) is a common complication of ST-segment elevation myocardial infarction (STEMI) and can lead to adverse cardiovascular events. This is a non-randomized, observational, prospective study of STEMI patients with multivessel disease who underwent primary PCI, grouped based on whether they underwent balloon pre-dilatation stenting or direct stenting of the culprit lesion. Coronary physiology measurements were performed 3 months post-PCI including coronary flow reserve (CFR) and index of microcirculatory resistance (IMR) measurements at the culprit vessel. The primary endpoint was the prevalence of CMD at 3 months, defined as IMR ≥ 25 or CFR < 2.0 with a normal fractional flow reserve. Secondary endpoints included major adverse cardiovascular events (MACE) at 12 months. Two hundred ten patients were enrolled; most were men, 125 (59.5%), with a median age of 65 years. One hundred twelve (53.2%) underwent balloon pre-dilatation before stenting, and 98 (46.7%) underwent direct stenting. The prevalence of CMD at 3 months was lower in the direct stenting group than in the balloon pre-dilatation stenting group (12.24% vs. 40.18%; *p* < 0.001). Aspiration thrombectomy and administration of intracoronary glycoprotein IIb/IIIa inhibitors were associated with lower odds of CMD (OR = 0.175, *p* = 0.001 and OR = 0.113, *p* = 0.001, respectively). Notably, MACE in patients who underwent direct stenting was lower than in those who underwent balloon pre-dilatation before stenting (14.29% vs. 26.79%; *p* = 0.040). In STEMI patients with multivessel disease, direct stenting of the culprit lesion, aspiration thrombectomy and administration of intracoronary glycoprotein IIb/IIIa inhibitors were associated with a lower prevalence of CMD at 3 months and lower incidence of MACE at 12 months compared with balloon pre-dilatation stenting.

This trial is registered at https://ichgcp.net/clinical-trials-registry/NCT05406297.

## Introduction

Prompt revascularization with primary percutaneous coronary intervention (PCI) of the occluded epicardial coronary artery is the standard of care for patients with ST-segment elevation acute myocardial infarction (STEMI)^1^. Although contemporary PCI can achieve patency in most cases, the restoration of coronary microcirculation and, subsequently, myocardial perfusion did not recover up to 50% of the STEMI patients^2^. Up to 15.5% of STEMI patients continue to experience persistent anginal symptoms after primary PCI, and the presence of coronary microvascular dysfunction (CMD) has been independently associated with worse angina status in this patient population^3^.

CMD is a term used to describe a range of anatomical and functional changes within the coronary microcirculation that reduce coronary blood flow to cardiomyocytes and result in myocardial ischemia^4^. CMD can be diagnosed using a variety of invasive and non-invasive techniques^5^. The thermodilution-based assessment of coronary flow reserve (CFR) and index of microcirculatory resistance (IMR) with a pressure–temperature sensor coronary guidewire is a highly reproducible, easily performed, and is the most common invasive method for diagnosing CMD^6,7^. Previous studies have shown a robust association between CFR, IMR and poor outcomes in patients with STEMI^8^.

The pathogenesis of CMD following revascularization in STEMI patients remains unclear. Recent hypotheses have linked CMD to distal embolization of thrombotic debris following mechanical fragmentation of the occlusion during pre-dilatation^9^. The presence of CMD is related to larger infarct size, higher peak enzyme level, and worse prognosis^10^ Direct stenting of the culprit occlusion, without pre-dilatation, may attenuate downstream embolization and, by extension, CMD^11^.

The purpose of this research was to determine whether there is a correlation between the technique of primary PCI used and the prevalence of CMD after reperfusion.

## Methods

### Study design

This is a prospective, single-blinded, non-randomized, observational, single-center trial conducted in the Hospital of Lithuanian University of Health Sciences Kauno klinikos, Kaunas, Lithuania. This study enrolled STEMI patients with multivessel coronary artery disease who underwent primary PCI according to the European Society of Cardiology (ESC) guidelines^12^. The PCI strategy was left to the discretion of the treating interventional cardiologist. Three months later, the patients underwent staged revascularization, followed by an invasive coronary physiology evaluation for CMD.

### Study inclusion and exclusion criteria

Participants were adults aged 40 years and older with STEMI (ST elevation ≥ 2 mm in ≥ 2 contiguous chest leads or ≥ 1 mm in ≥ 2 contiguous limb leads) who had received dual antiplatelet therapy (acetylsalicylic acid 300 mg and ticagrelor 180 mg or clopidogrel 600 mg) at least 30 min prior to primary PCI of the culprit vessel and subsequently underwent staged PCI of the non-culprit vessel 3 months later.

To exclude the potential influence of pre-existing microvascular obstruction, patients with a history of acute coronary syndrome were excluded. Patients who did not have a non-culprit coronary lesion and thus did not require a follow-up angiogram were also excluded. Patients with a serious comorbid illness such as sepsis, autoimmune disease, end-stage liver disease, end-stage renal failure, or solid organ cancer were excluded. Patients with severe valvular heart disease were excluded for significantly variable coronary physiology, and those with coronary artery bypass grafts were excluded because of altered coronary circulation^13^. Patients who underwent primary fibrinolysis, were allergic to contrast media, or were unable to tolerate adenosine triphosphate were also excluded.

### Primary percutaneous coronary intervention

Primary PCI was performed using 6-Fr guiding catheters via radial or femoral arterial approaches. Patients were anticoagulated with a heparin bolus (70–100 U/kg) administered either intravenously, or directly into the coronary artery via the guiding catheter. The route of heparin administration was up to the discretion of the operator and was recorded prospectively. Two interventional cardiologists blinded to treatment allocation and study data independently assessed angiographic variables such as the TIMI flow score at baseline and at the completion of the primary PCI procedure. The treating operator independently determined whether to pursue balloon pre-dilatation stenting (balloon pre-dilatation followed by stenting) or direct stenting (stenting without balloon pre-dilatation). In accordance with standard procedural practices, all patients in both groups underwent post-dilation following stent implantation. This routine step ensures optimal stent apposition and expansion, minimizing the risks associated with potential stent under-expansion or malapposition. Iopromide (Ultravist, Bayer HealthCare Pharmaceuticals, Leverkusen, Germany) was used as the contrast agent. The study team documented information such as stent diameter and length, maximum inflation pressure, and the amount of contrast agent. The decision to use an aspiration catheter (Thrombuster II manual thrombus aspiration catheter, Kaneka Inc., Osaka, Japan) during the primary PCI was determined by the treating physician and was prospectively documented. Per institutional guidelines, an intracoronary glycoprotein IIb/IIIa inhibitor was administered if a TIMI flow score of 3 was not achieved after epicardial revascularization of the culprit artery during the initial presentation with STEMI. Relative contraindications included age > 80 years, a low hemoglobin, history of hemorrhagic stroke or bleeding requiring blood transfusion; the decision to administer glycoprotein IIb/IIIa inhibitor was ultimately determined by the treating physician and prospectively documented.

### Coronary physiology assessment

All coronary physiology measurements were performed 3 months after the STEMI by an experienced interventionalist who was blinded to the revascularization technique employed during primary PCI. CFR, fractional flow reserve (FFR), and IMR were assessed using the CoroFlow system (Coroventis Research AB, Uppsala, Sweden). After undergoing successful staged PCI, nitroglycerin was administered through the guiding catheter, and a coronary pressure/temperature sensor-tipped guidewire (Pressure Wire X; Abbott Vascular, Santa Clara, CA, United States) was equalized to the guide catheter pressure with the pressure sensor positioned at the tip of the catheter at the aortic sinus, then advanced to the distal two-thirds of the infarct related artery. Maximal hyperemia was induced by repeated intracoronary adenosine boluses. After achieving maximal hyperemia, three milliliters of normal saline were administered through the guiding catheter and IMR was calculated. If the measurements obtained from the first three administrations were inconsistent, the measurements were repeated to ensure accuracy CFR was determined as the difference between the baseline and hyperemic mean transit time (T_mn_). IMR was computed by multiplying the distal coronary pressure during maximal hyperemia by the hyperemic T_mn_. The ratio of mean distal (d) to mean proximal (p) coronary artery pressure (P) during maximal hyperemia was used to calculate FFR (FFR = Pd/Pa).

### Data collection and echocardiographic imaging

Patient demographics, medical history, clinical course, laboratory values, angiographic characteristics, and follow-up data were collected prospectively. Left ventricular ejection fraction (LVEF) was assessed by acquiring 2-dimensional and 3-dimensional images using ultrasound (EPIQ 7, Phillips Ultrasound, Inc., Washington, USA) at 24 h and 1-year post-STEMI. These images were acquired by a trained cardiovascular imaging technician who was blinded to the study data and followed the guidelines established by the European Association of Cardiovascular Imaging (EACVI)^14^.

### Study endpoints

The primary endpoint was the presence of CMD three months after STEMI. The secondary endpoint was the rate of major adverse cardiovascular events (MACE) within 12 months of follow-up.

### Definitions

STEMI was defined according to the fourth universal definition of myocardial infarction^15^. Door-to-wire time was defined as the time (in minutes) from the first medical contact at the facility to the time of advancement of the PCI wire. Dyslipidemia was defined as a fasting total cholesterol level > 70 mg/dl (1.8 mmol/l) or the use of lipid-lowering medications^16^. Hypertension was defined as a blood pressure ≥ 140/90 mmHg or the use of blood pressure-lowering medication^17^. Diabetes mellitus was defined as a fasting plasma glucose level ≥ 7.0 mmol/l, or the use of blood glucose-lowering medication^18^. MACE was defined as the composite endpoint of cardiovascular death, non-fatal myocardial infarction, target vessel revascularization, recurrent hospitalization due to decompensated heart failure, and stroke (ischemic or hemorrhagic). Renal function was assessed by calculating the glomerular filtration rate using the Cockcroft-Gault equation.

Successful PCI was defined as the implantation of a second-generation drug-eluting stent to the target lesions, resulting in visual reduction of the lesion to less than 20% stenosis, and restoration of coronary blood flow equivalent to both TIMI 2 and TIMI 3 Flow levels. Normal values for FFR, CFR, and IMR were defined as > 0.80, ≥ 2.0, and < 25 U, respectively^19,20^. Microcirculatory dysfunction was defined as IMR ≥ 25 or a CFR < 2.0^7,19–21^. While an IMR > 40 immediately after PCI in STEMI patients has been shown to predict MACE, coronary physiology measurements in this study were performed 3 months after PCI, hence this threshold was not applicable^22^. Nevertheless, a sensitivity analysis examining the incidence of MACE at 12 month follow-up in patients with an IMR < 25, 25 ≤ IMR ≤ 40, and IMR > 40 was performed (Supplemental Fig. 1).

### Statistical analysis

Continuous variables were determined to be skewed and therefore were presented as median values with quartile ranges. Categorical variables were presented as frequency and percentage. Wilcoxon Rank Sum, Chi-Square, or Fisher's Exact tests were used to assess baseline differences and outcomes between the study groups, as appropriate. Stepwise selection was used to create multivariable logistic regression models to investigate procedural factors associated with CMD. Kaplan–Meier analysis was used to assess MACE-free survival rates, and differences were evaluated using the log-rank test. A probability level of p < α (where α is the significance level set at 0.05) was assumed to determine statistical significance. Data processing was performed using IBM SPSS Statistics 27.

### Ethics approval and consent to participate

We conducted this study in compliance with the ethical standards of the Regional Bioethics Committee of Kaunas, Lithuania (the permission number is BE-2-5) and the World Medical Association Declaration of Helsinki on Ethical Principles for Medical Research Involving Human Subjects. Clinical Trials registration number: NCT05406297, concurrently registered. All subjects gave their informed consent to participate, and an information letter was given to them.

## Results

### Study population characteristics

This study enrolled 210 patients, of whom 98 patients (46.7%) underwent direct stenting and 112 (53.2%) underwent balloon pre-dilatation before stenting. The median age of the patients was 65 years, falling within an interquartile range of 58 to 76 years. Of the total, 125 patients, accounting for 59.5%, were male. This gender distribution was similar among those who underwent direct stenting and those who received balloon pre-dilatation prior to stenting. Both groups had similar body mass indices, body surface areas, and culprit vessels. Fifty-one (24.3%) patients had diabetes, and 109 (51.9%) patients were current or former smokers, with similar distributions between the two groups. Other risk factors, including arterial hypertension, dyslipidemia, Killip classification, CHADS2-VASc score, and history of heavy alcohol use, stroke, or coronary artery disease, were also similar between the two groups (Table 1).

**Table 1:** Characteristics of ST-elevation myocardial infarction patients classified by percutaneous coronary intervention technique.

Characteristic	Overall (n = 210)	Direct stenting (n = 98)	Balloon pre-dilatation stenting (n = 112)	P-value
Sex (Female)	85 (40.48%)	35 (35.71%)	50 (44.64%)	0.240
Age (years)	65.0 [58, 76]	67.0 [58.25, 76.0]	63.50 [56.0, 75.0]	0.477
Body mass index (kg/m^2^)	27.39 [24.56, 30.69]	27.71 [25.32, 30.60]	26.38 [24.29, 31.10]	0.443
Body surface area (m^2^)	1.93 [1.81, 2.10]	1.94 [1.81, 2.10]	1.92 [1.83, 2.12]	0.768
Primary diagnosis				
Anterior STEMI	116 (55.24%)	53 (54.08%)	63 (56.25%)	0.860
Inferior STEMI	94 (44.76%)	45 (45.92%)	49 (43.75%)	
Arterial hypertension	123 (58.57%)	58 (59.18%)	65 (58.04%)	0.978
History of coronary artery disease	59 (28.10%)	27 (27.55%)	32 (28.57%)	0.992
History of PCI	26 (12.38%)	11 (11.22%)	15 (13.39%)	0.790
History of stroke	27 (12.86%)	11 (11.22%)	15 (13.39%)	0.649
History of diabetes mellitus	51 (24.29%)	21 (21.43%)	30 (26.79%)	0.458
History of dyslipidemia	119 (56.67%)	60 (61.22%)	59 (52.68%)	0.268
Smoker (former/current)	109 (51.90%)	51 (52.04%)	58 (51.79%)	1
History of alcohol abuse	20 (9.52%)	8 (8.16%)	12 (10.71%)	0.695
Baseline CHADS2-VASc score	3 [2, 4]	3 [2, 4]	3 [2, 4]	0.851
KILLIP class				
I	62 (29.52%)	27 (27.55%)	35 (31.25%)	0.519
II	111 (52.86%)	53 (54.08%)	58 (51.79%)	
III	27 (12.86%)	15 (15.31%)	12 (10.71%)	
IV	10 (4.76%)	3 (3.06%)	7 (6.25%)	

*STEMI* ST elevation myocardial infarction, *PCI* percutaneous coronary intervention.

### Laboratory and echocardiographic findings

Complete blood counts, creatinine clearance, initial troponin and peak troponin levels were similar between the two groups. The direct stenting group had a slightly higher total cholesterol (5.1 mmol/l vs 4.4 mmol/l; *p* = 0.035) and low-density lipoprotein cholesterol (3.5 mmol/l vs 3.2 mmol/l; *p* = 0.047). The creatinine clearance rate (40.5 ml/min vs 38.2 ml/min; *p* = 0.121) and initial troponin levels were similar between the two study groups. There was no difference in LVEF after primary PCI between the two groups (42.0% vs. 40%; *p* = 0.914), while the LVEF of the direct stenting group was higher at 12 months (48% vs. 45%; *p* = 0.003) (Table 2).

**Table 2:** Laboratory and echocardiographic parameters of patients with ST-elevation myocardial infarction, categorized by percutaneous coronary intervention technique.

Parameters	Overall (n = 210)	Direct stenting (n = 98)	Balloon pre-dilatation stenting (n = 112)	P-value
*Laboratory test*				
Hemoglobin (g/l)	136.0 [119.0, 148.0]	137.0 [121.0, 146.8]	135.0 [118.0, 148.3]	0.631
White blood cell count (10^9^/l)	9.86 [8.22, 12.09]	9.78 [8.09, 12.07]	10.05 [8.38, 12.10]	0.742
Platelets (× 10^9^/l)	240.5 [204.0, 273.0]	242.0 [212.5, 273.8]	240.0 [200.0, 271.5]	0.635
Total cholesterol (mmol/l)	4.64 [3.75, 5.79]	5.06 [4.07, 6.01]	4.43 [3.60, 5.57]	0.035
Low-density lipoprotein (mmol/l)	3.26 [2.37, 4.31]	3.51 [2.66, 4.46]	3.17 [2.23, 4.08]	0.047
High-density lipoprotein (mmol/l)	1.12 [0.92, 1.35]	1.11 [0.94, 1.36]	1.13 [0.91, 1.32]	0.568
Triglycerides (mmol/l)	1.16 [0.82, 1.65]	1.11 [0.82, 1.72]	1.17 [0.85, 1.57]	0.896
Creatinine clearance (mL/min)	39.5 [34.95, 47.5]	40.45 [35.28, 48.68]	38.20 [34.80, 47.10]	0.121
Basal troponin I (µg/l)	2.19 [0.81, 3.71]	2.22 [0.96, 3.89]	2.18 [0.76, 3.29]	0.427
Peak troponin I (µg/l)	45.0 [27.0, 64.0]	42.0 [26.25, 67.75]	46.0 [28.0, 62.0]	0.830
High-sensitivity C-reactive protein (mg/l)	3.80 [1.85, 10.52]	4.22 [1.85, 10.06]	3.66 [1.94, 10.77]	0.872
*Echocardiographic parameters*				
Post-PCI left ventricular ejection fraction (%)	40.0 [36.25, 45.75]	42.0 [38.5, 45.0]	40.0 [35.75, 46.25]	0.913
12-month left ventricular ejection fraction (%)	45.0 [40.0, 50.0]	48.0 [40.0, 55.0]	45.0 [35.0, 50.0]	0.003

*CMD* coronary microvascular dysfunction, *PCI* percutaneous coronary intervention.

### Procedural characteristics and coronary physiology findings

Pain-to-door time (278.5 min vs. 348 min; *p* = 0.166) and door-to-balloon time (39.0 min vs. 41.5 min; *p* = 0.318) TIMI flow before and after PCI were similar between the two groups. The left anterior descending artery was the culprit artery in 118 (56.2%) patients at a similar rate in both groups (53 (54.1%) vs 65 (58.0%); *p* = 0.771). Intracoronary heparin was utilized in 104 (49.5%) patients, intracoronary glycoprotein IIb/IIIa inhibitor was utilized in 46 (21.9%) patients, and aspiration thrombectomy was performed on 52 (24.8%) patients. The rates of these interventions were similar between the groups undergoing direct stenting and balloon pre-dilatation before stenting: 57.1% vs. 42.9%; *p* = 0.054, 21.4% vs. 22.3%; *p* = 1, and 25.5% vs. 24.1%; *p* = 0.940, respectively. Furthermore, there were no significant differences in the study groups regarding contrast dose, stent diameter and length, or maximal inflation pressure (Table 3).

**Table 3:** Coronary angiography and physiology parameters of ST-elevation myocardial infarction patients, categorized by percutaneous coronary intervention technique.

Parameters	Overall (n = 210)	Direct stenting (n = 98)	Balloon pre-dilatation stenting (n = 112)	P-value
*Angiographic*				
Pain-to-door time (minutes)	314 [107.75, 592.25]	348.0 [112.0, 677.75]	278.5 [108.75, 460.25]	0.166
Door‐to‐balloon (minutes)	40 [29.25, 52.0]	41.5 [31.0, 51.75]	39.0 [29.0, 52.25]	0.318
Pre-PCI TIMI flow				
0	130 (61.90%)	55 (56.12%)	75 (66.96%)	0.305
1	8 (3.81%)	3 (3.06%)	5 (4.46%)	
2	44 (20.95%)	25 (25.51%)	19 (16.96%)	
3	28 (13.33%)	15 (15.31%)	13 (11.61%)	
Post-PCI TIMI flow				
0	2 (0.95%)	1 (1.02%)	1 (0.89%)	0.824
1	1 (0.48%)	0 (0.0%)	1 (0.89%)	
2	22 (10.48%)	10 (10.20%)	12 (10.71%)	
3	185 (88.1%)	87 (88.78%)	98 (87.50%)	
Culprit vessel				
Left anterior descending artery	118 (56.19%)	53 (54.08%)	65 (58.04%)	0.771
Circumflex artery	49 (23.33%)	25 (25.51%)	24 (21.43%)	
Right coronary artery	43 (20.48%)	20 (20.41%)	23 (20.54%)	
Number of diseased vessels				
2-Vessel disease	123 (58.57%)	56 (57.14%)	67 (59.82%)	0.801
3-Vessel disease	87 (41.43%)	42 (42.86%)	45 (40.18%)	
*Intracoronary interventions*				
Intracoronary heparin infusion	104 (49.52%)	56.0 (57.14%)	48.0 (42.86%)	0.054
Intracoronary glycoprotein IIb/IIIa inhibitor	46 (21.9%)	21.0 (21.43%)	25.0 (22.32%)	1
Aspiration thrombectomy	52 (24.76%)	25.0 (25.51%)	27.0 (24.11%)	0.940
Stent diameter (millimeters)	3.0 [3.0, 3.5]	3.0 [3.0, 3.5]	3.0 [3.0, 3.5]	0.699
Stent length (millimeters)	24.0 [19.0, 26.0]	24.0 [19.0, 26.0]	24.0 [19.0, 26.0]	0.293
Maximal stent pressure (atm)	14.0 [14.0, 16.0]	15.0 [14.0, 17.0]	14.0 [13.0, 16.0]	0.160
Contrast dose (milliliters)	100.0 [90.0, 110.0]	100.0 [90.0, 110.0]	100.0 [90.0, 111.25]	0.262
*Coronary physiology at 3-month follow-up*				
Coronary flow reserve	2.81 [2.54, 2.98]	2.87 [2.65, 3.14]	2.70 [2.16, 2.95]	0.003
Fractional flow reserve	0.92 [0.87, 0.97]	0.92 [0.87, 0.97]	0.92 [0.86, 0.97]	0.452
Index of microvascular resistance	20 [15.0, 29.0]	19.5 [14.0, 22.0]	22.0 [15.0, 42.0]	0.001
Coronary microvascular dysfunction	57 (27.14%)	12.0 (12.24%)	45.0 (40.18%)	< .001

*PCI* percutaneous coronary intervention.

**Figure 1:**
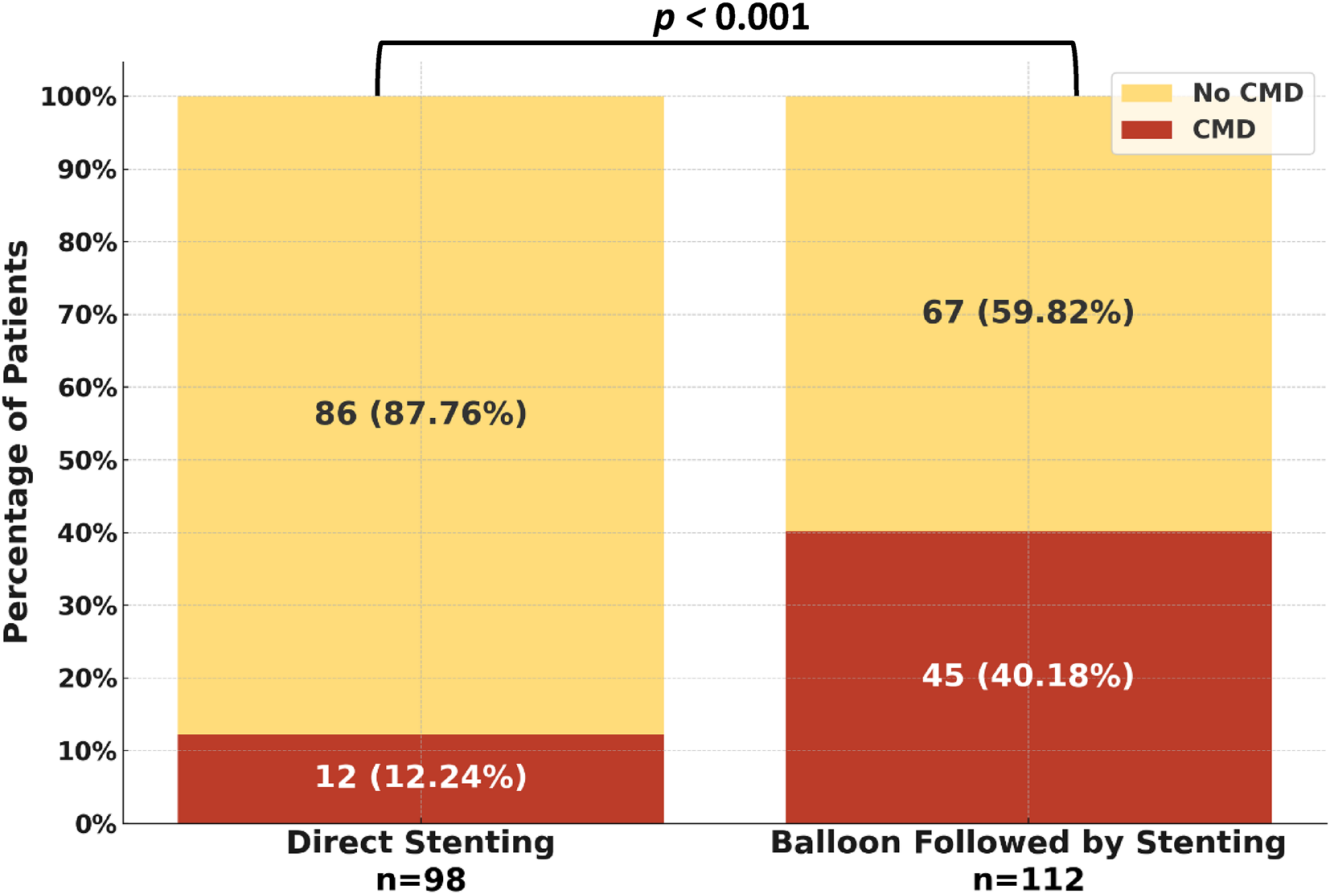
Prevalence of coronary microvascular dysfunction displayed by percutaneous coronary intervention technique.

Three months post-primary PCI, the FFR values exhibited no significant difference between patients who underwent direct stenting and those who were subject to balloon pre-dilatation before stenting (0.92 vs 0.92; *p* = 0.452). Conversely, CFR values demonstrated a significant increase (2.87 vs 2.70; *p* < 0.001), and the IMR values displayed a notable decrease (19.5 vs 22.0; *p* = 0.001) in the patients who underwent direct stenting compared to those who underwent balloon pre-dilatation. Furthermore, there was a lower prevalence of CMD in the direct stenting group (12.2% vs 40.2%; *p* < 0.001) (Table 3, Fig. 1).

### Major adverse cardiovascular events at 12 months

**Figure 2:**
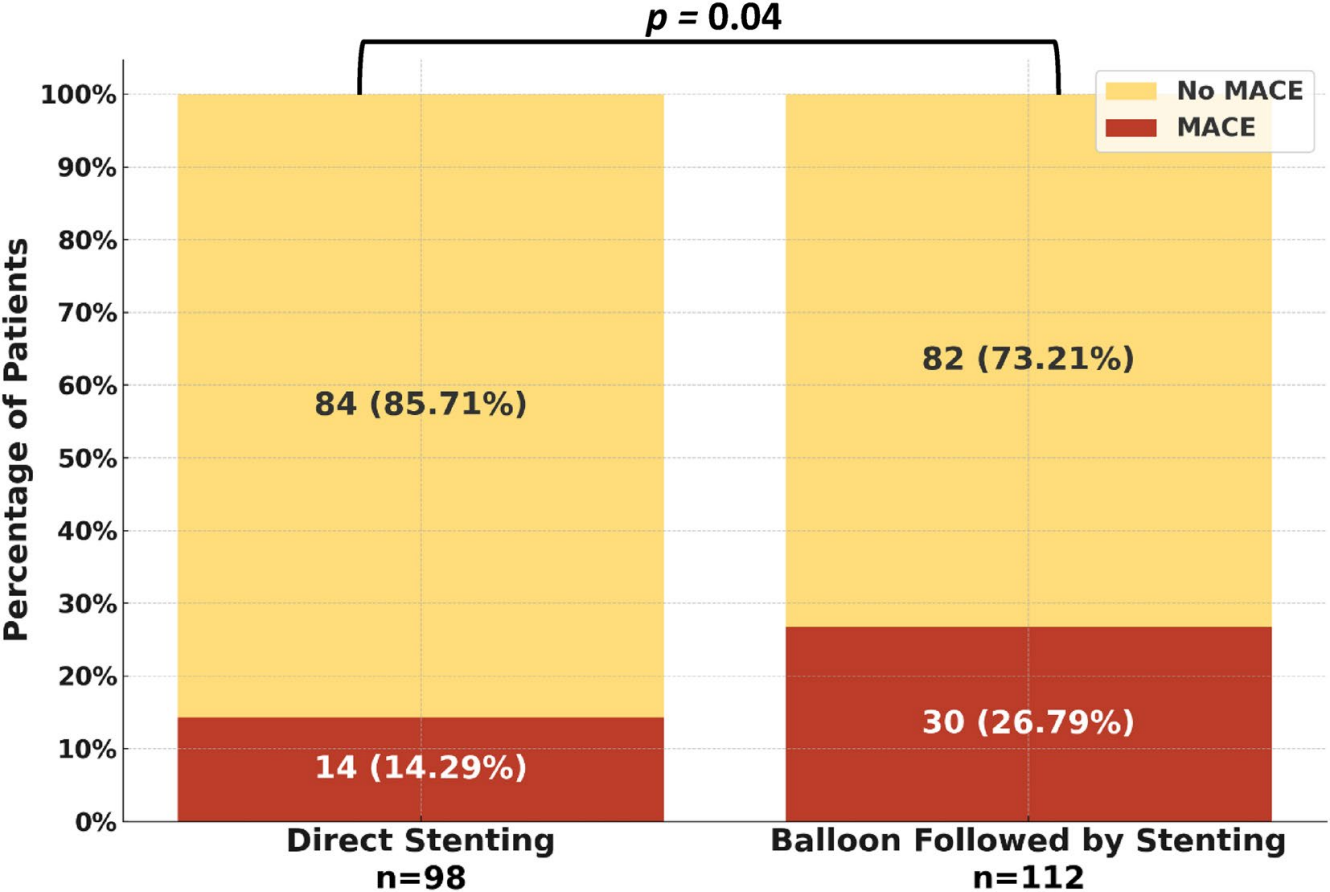
Rates of major adverse cardiac events displayed by percutaneous coronary intervention technique.

**Figure 3:**
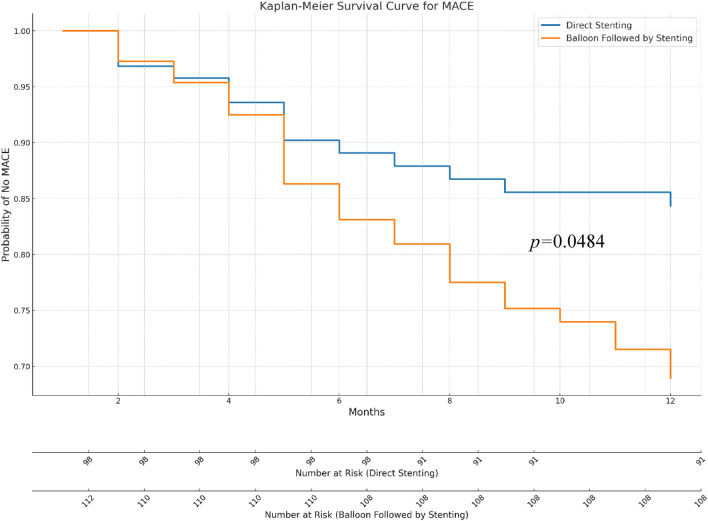
Kaplan–Meier event-free survival curve for occurrence of major adverse cardiac events grouped by percutaneous coronary intervention technique.

At 12 month follow-up, the incidence of MACE in patients who underwent direct stenting was lower than in those who underwent balloon pre-dilatation before stenting (14.3% vs. 26.8%; *p* = 0.040) (Table 4, Fig. 2). This difference was driven largely by a decreased incidence of stroke in patients who underwent direct stenting (0% vs 6.3%; *p* = 0.033). The Kaplan–Meier curve revealed an increased incidence of MACE in patients who underwent balloon pre-dilatation stenting (log-rank *p* = 0.048), most noticeably starting at 6 months post-PCI (Fig. 3).

**Table 4:** Twelve-month clinical outcomes of patients presenting with ST-elevation myocardial infarction, categorized by percutaneous coronary intervention technique.

	Overall (n = 210)	Direct stenting (n = 98)	Balloon pre-dilatation stenting (n = 112)	P-value
Ischemic or hemorrhagic stroke	7 (3.33%)	0 (0%)	7 (6.25%)	0.033
Nonfatal MI	10 (4.76%)	3 (3.06%)	7 (6.25%)	0.448
Cardiovascular death	6 (2.86%)	1 (1.02%)	5 (4.46%)	0.280
Target vessel revascularization	10 (4.76%)	6 (6.12%)	4 (3.57%)	0.588
Decompensated HF requiring hospitalization	16 (7.62%)	5 (5.10%)	11 (9.82%)	0.305
MACE	44 (20.95%)	14 (14.29%)	30 (26.79%)	0.040

*MI* myocardial infarction, *HF* heart failure, *MACE* the composite of stroke, nonfatal myocardial infarction, revascularization, heart failure hospitalization, and cardiovascular death.

A sensitivity analysis examining the incidence of MACE at 12 months according to IMR category revealed a lower incidence of MACE in patients with an IMR < 25, when compared to those with an 25 ≤ IMR ≤ 40 (*p* < 0.001) and those with an IMR > 40 (*p* < 0.001). These was no difference in the incidence of MACE at 12 months in patients with an 25 ≤ IMR ≤ 40 and those with an IMR > 40 (*p* = 0.352) (Supplemental Fig. 1).

### Multivariable logistic analysis

**Figure 4:**
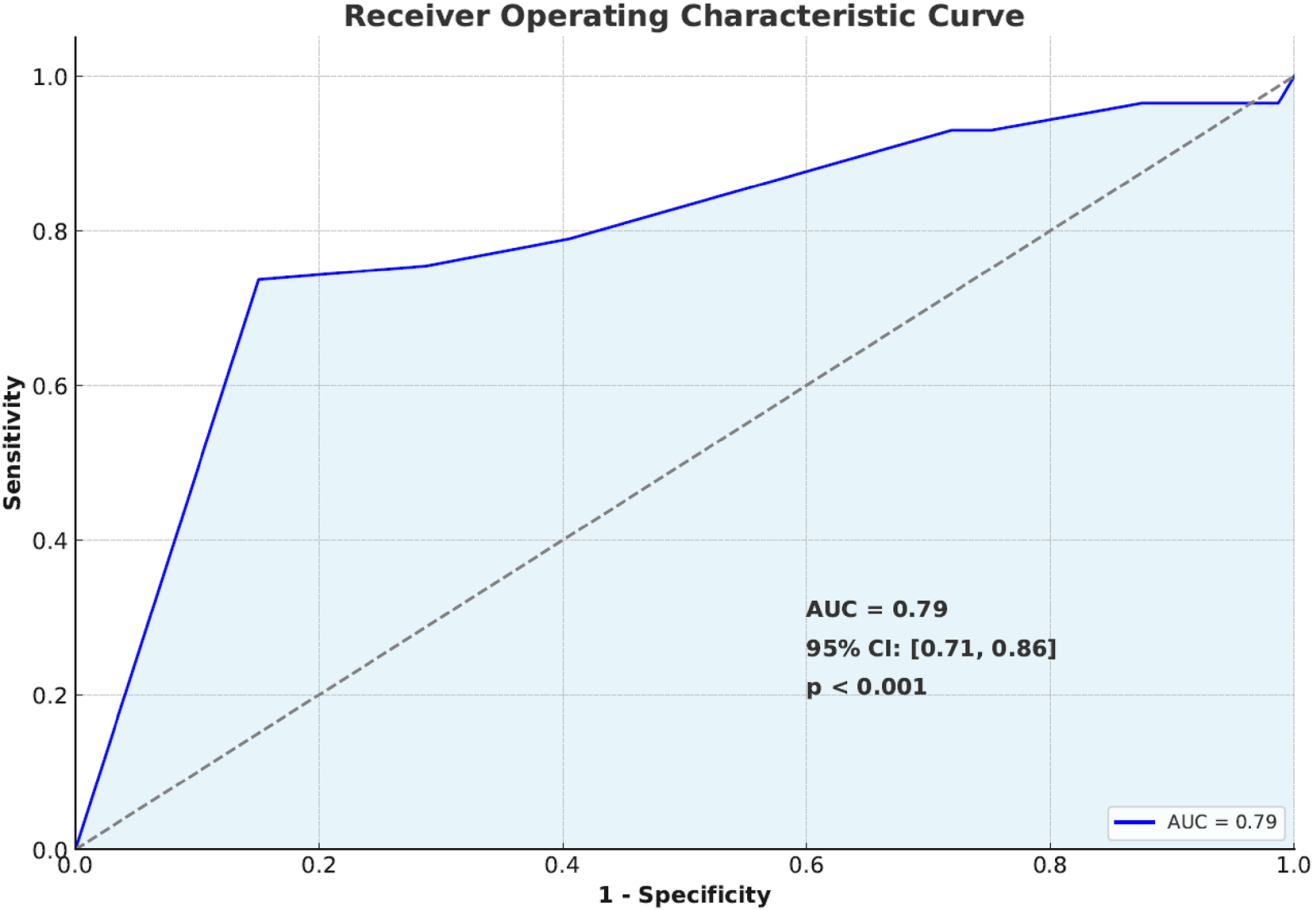
Receiver Operating Characteristic curve for the model of coronary microvascular dysfunction in ST-elevation myocardial infarction patients. *AUC* area under the receiver operating characteristic curve.

**Figure 5:**
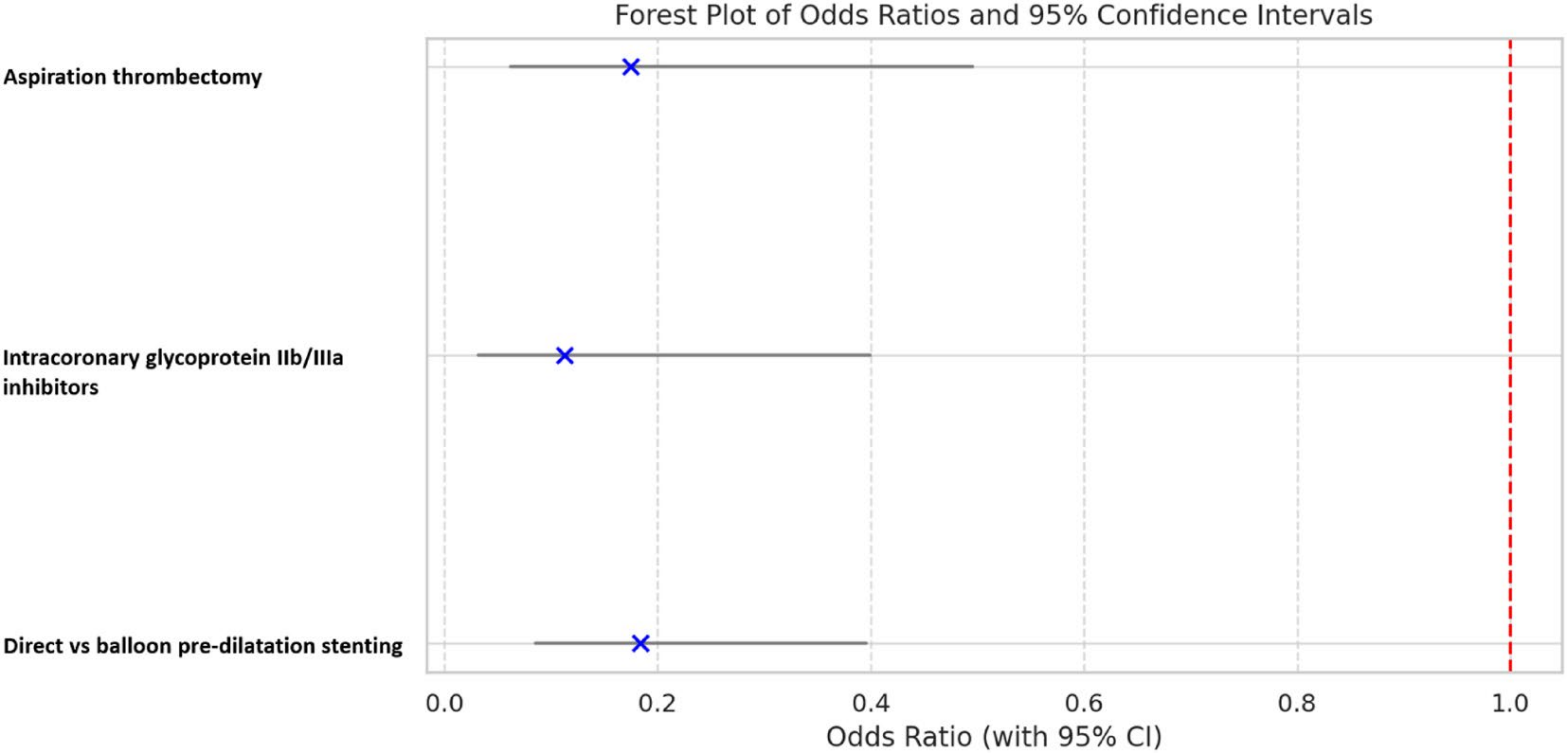
Forest plot for the outcome of coronary microvascular dysfunction.

The multivariable logistic regression analysis revealed that direct stenting, aspiration thrombectomy, and utilization of glycoprotein IIb/IIIa jointly yielded a receiver operator characteristic area under the curve of 0.79 (0.71–0.86), indicating a good predictive ability in the binary logistic multivariable analysis (Fig. 4). In this particular model, we were able to determine that the use of direct stenting as opposed to balloon pre-dilatation before stenting, the utilization of aspiration thrombectomy, and the administration of intracoronary glycoprotein IIb/IIIa were associated with decreased odds of CMD (odds ratio (OR): 0.184, 95% confidence interval (CI): 0.085–0.396, *p* < 0.001), (OR: 0.175, 95% CI: 0.062–0.495, *p* = 0.001), and (OR: 0.113, 95% CI: 0.032–0.399, *p* = 0.001) (respectively (Table 5, Fig. 5).

**Table 5:** Multivariable binary logistic analysis for prediction of coronary microvascular dysfunction after ST-segment elevation myocardial infarction event.

Effect	Odds ratio	95% Confidence limits		P-value
Direct stenting vs balloon pre-dilatation stenting	0.184	0.085	0.396	< .001
Aspiration thrombectomy	0.175	0.062	0.495	0.001
Glycoprotein IIb/IIIa	0.113	0.032	0.399	0.001

## Discussion

This prospective, single-blinded, cohort study is one of the largest studies examining the relationship between PCI technique and CMD in STEMI patients. Direct stenting was associated with a lower prevalence of CMD and adverse cardiac events when compared to balloon pre-dilatation before stenting. Both aspiration thrombectomy and administration of glycoprotein IIb/IIIa inhibitors were associated with a lower prevalence of CMD. Unlike prior studies that assessed CMD during the acute phase of STEMI, we performed coronary physiology testing three months after STEMI^23,24^ because of altered coronary physiology during the acute phase of STEMI^25–27^. Ríos-Navarro et al., in their experimental study, demonstrated a near-complete resolution of CMD 30 days post-reperfusion^26^. This hypothesis found further empirical validation in the research conducted by Demirkiran et al., wherein a marked optimization of CMD indices was observed within the 30-day post-STEMI period. Specifically, data demonstrated a decrement in IMR metrics from a pre-established 38.8 to a subsequent 25.6, concomitant with an augmentation in CFR from an initial measurement of 2.16 to a later 3.77^27^. Current guidelines establish primary PCI as the gold standard for treating STEMI patients, but do not specify whether to pursue direct stenting or balloon predilatation^1^.

In this study, multivariable logistic analysis showed that direct stenting was associated with approximately fivefold lower odds of CMD compared with balloon pre-dilatation stenting (Table 5, Fig. 5). There are several potential explanations for this observation. First, balloon manipulation within the culprit lesion may cause distal embolization of fragmented thrombus or atheromatous debris, aggravating microvascular occlusion and leading to prolonged myocardial ischemia^28^. Webb and colleagues observed that in saphenous venous graft lesions, direct stenting resulted in less distal embolization than predilatation followed by stenting, likely because thrombus and friable material were entrapped behind the stent struts^29^. Kalayci and colleagues similarly found that STEMI patients treated with direct stenting were less likely to exhibit visible distal embolization (4.4% vs 7.4%; *p* = *0.014*) and were more likely to have complete resolution of ST segment elevation (68.9% vs 59.6%; *p* < *0.001*) than those treated with balloon pre-dilatation stenting^30^. Second, inflation of the balloon within the culprit lesion may release atherogenic plaque components which further activate the coagulation cascade^31^. Third, predilatation may result in arterial wall endothelial dissection and subsequent rapid thrombosis. Fourth, pre-dilatation may propagate endothelial damage, triggering an inflammatory response and limiting the appropriate endothelialization of the stented vessel, thereby increasing the risk of stent thrombosis or neointimal hyperplasia^31^. Direct stenting also has the potential to reduce radiation exposure and healthcare expenditures by reducing procedure time^32^.

However, direct stenting also has limitations, including difficulty estimating the caliber of the coronary artery, which may result in inadequate stent expansion, difficulty or failure to deliver or optimally position the stent due to inadequate visualization of the lesion margins^33^.

Previous studies investigating the relationship between PCI techniques and CMD have shown contradictory results. He and colleagues investigated the impact of stenting technique on CMD and did not find that direct stenting had any advantages over balloon predilatation. However, the authors utilized cardiac magnetic resonance (CMR) rather than invasive coronary physiology to assess CMD. Further, they performed CMR 1 week after STEMI, which may be too early to obtain a reliable assessment of coronary microcirculation^26,34^. Kim and colleagues failed to demonstrate any impact of PCI technique on microcirculation. While this study was randomized, it only included 38 patients in each arm, coronary physiology was ascertained immediately after primary PCI, and only IMR was used to assess CMD, rather than both IMR and CFR^23^. Several other randomized controlled trials investigating direct stenting and its effect on myocardial perfusion have been conducted, but are outdated (conducted between the late 1990s and early 2000s) and do not reflect current clinical practice or modern techniques to assess CMD^35–39^.

A study by Scarparo and colleagues found that among STEMI patients who had a higher thrombus burden (TIMI grade flow 0–1), those who were treated with direct stenting had a lower incidence of all-cause mortality at 15 years (hazard ratio (HR) 0.65, 95% CI 0.50–0.84, *p* = *0.001*) and MACE at 10 years (HR 0.71, 95% CI 0.55–0.92, *p* = *0.010*), when compared with those who were treated with balloon pre-dilatation before stenting^40^. McCormick and colleagues found that balloon pre-dilatation stenting was independently associated with one year mortality OR 2.42, 95 CI 1.08–5.45, *p* = *0.032*) compared with direct stenting. Neither study was randomized^41^. A randomized study by Cuisset and colleagues found a lower IMR with direct stenting compared with balloon pre-dilatation before stenting (13 ± 3 vs. 24 ± 14; *p* < *0.01*); however, this study was small (50 patients) and only included patients with stable angina^42^.

The present study also revealed that aspiration thrombectomy during primary PCI was associated with an approximately fivefold decrease in the prevalence of CMD (Table 5, Fig. 5). The use of an aspiration thrombectomy catheter during primary PCI is still being debated in the medical community, with investigations yielding contradictory data^24,43–45^. According to the Thrombectomy Trialists Collaboration study, direct stenting with aspiration thrombectomy during primary PCI did not enhance clinical outcomes or myocardial reperfusion parameters^43^. This is appropriately reflected in the current guidelines, which state that routine use of aspiration thrombectomy is not encouraged^1^. However, the Thrombectomy Trialists Collaboration study used myocardial blush to assess for CMD, rather than invasive thermodilution, CMR, or another quantifiable physiologic index. Hoole and colleagues conducted a randomized clinical pilot trial in which they performed a series of IMR measurements during different stages of primary PCI, followed by CMR analysis at 24 h and three-month follow-up. They found a trend toward less microcirculatory damage in patients who underwent aspiration thrombectomy; however, this did not reach statistical significance. The authors did acknowledge that the results should be interpreted with caution as only 26 patients were included in the CMR analysis, resulting in an underpowered study. Furthermore, IMR was only obtained during primary PCI, which may not be reliable because of altered coronary physiology during STEMI^24^.

The MUltidevice Thrombectomy in Acute ST-Segment Elevation Acute Myocardial Infarction trial, which was the largest randomized trial to evaluate the impact of aspiration thrombectomy on CMD, included 208 STEMI patients and assessed CMD via CMR at 3 months. The aspiration thrombectomy group had a lower prevalence of CMD (11.4% vs. 26.7%, *p* = 0.02)^46^. Similarly, the Thrombectomy With Export Catheter in Infarct-Related Artery During Primary Percutaneous Coronary Intervention—EXPIRA trial found that aspiration thrombectomy led to a smaller percentage of left-ventricular myocardium with microvascular obstruction (31.5% vs. 72.9%, *p* = 0.0005), when assessed by CMR during the acute phase of STEMI^47^. Another randomized trial conducted by Zajdel and colleagues found that aspiration thrombectomy was associated with less infarcted myocardium with microvascular obstruction on CMR at 6 months (9.0% vs. 26.9%, *p* = 0.009), although this analysis only included 45 patients^45^.

Finally, our study revealed that administration of an intracoronary glycoprotein IIb/IIIa inhibitor (eptifibatide) during primary PCI was associated with an approximately fivefold decreased risk of CMD (Table 5, Fig. 5). Intracoronary administration of glycoprotein IIb/IIIa inhibitors allows for higher drug concentrations, and therefore, increased drug activity in the target vessel^48^. Platelet aggregation, which plays a role in microvascular obstruction, is attenuated by glycoprotein IIb/IIIa inhibitors^49^. In a rodent model, administration of a glycoprotein IIb/IIIa inhibitor during STEMI preserved the structural and functional integrity of the microvascular endothelium via a process involving nitric oxide^50^. Akpek and colleagues conducted a randomized controlled trial and found that patients who received an intracoronary glycoprotein IIb/IIIa inhibitor had better TIMI flow after primary PCI compared with those who received placebo^51^.

## Limitations

The main limitation of the study was that it was an observational, not randomized, and single center study. However, prior randomized studies examining this topic have suffered from high rates of patient cross-over, limiting the interpretability of their results^23,52,37^. Despite the study's relatively small sample size (2010 patients), it is one of the largest prospective studies evaluating invasive CMD testing in patients presenting with STEMI. While the door-to-balloon times in the current study adhered to the ESC guidelines, we observed extended pain-to-door times^12^. This is often attributed to patient factors and a matter of public health awareness. However, in a sub-analysis, we demonstrated that the prevalence of CMD was lower in patients receiving direct stenting irrespective of their pain-to-door time (Supplemental Table 1). We were unable to assess the presence of CMD prior to STEMI and could only ascertain the prevalence of CMD at 3 months. Intracoronary glycoprotein IIb/IIIa inhibitors were administered if a TIMI flow score of 3 was not achieved after epicardial revascularization, which is a potential confounder in our analysis. Due to ethical concerns, our study only included patients with multivessel coronary artery disease who required staged PCI at 3-month follow-up, thus limiting the generalizability of our findings. While the study found that direct stenting was associated with a decreased incidence of MACE, this was driven by a reduction in the incidence of stroke. Intriguingly, prior studies have shown that patients with CMD have a higher prevalence of atrial fibrillation^8^. We hypothesized that these strokes might be cardioembolic in nature, given the increased rates of new-onset atrial fibrillation in patients who underwent balloon pre-dilatation. However, this trend was not statistically significant (15.18% vs 6.12%; *p* = 0.061), and larger studies will be needed to confirm our findings.

## Conclusions

In conclusion, our study suggests that direct stenting, aspiration thrombectomy, and intracoronary injection of glycoprotein IIb/IIIa inhibitors each is associated with a lower prevalence of CMD at 3 months and lower incidence of MACE at 12 months when compared with balloon pre-dilatation before stenting. Further research is needed to confirm our results in larger, randomized trials conducted across multiple institutions.

### Supplementary Information

Supplementary Table 1.

Supplementary Figure 1.

## Data Availability

The datasets used in this study are available from the corresponding author on reasonable request.
