# Supplementary material for: The impact of primary percutaneous coronary intervention strategies during ST-elevation myocardial infarction on the prevalence of coronary microvascular dysfunction
Source: Sci Rep. 2023 Nov 16;13:20094. doi: 10.1038/s41598-023-47343-x (PMC10654664; doi:10.1038/s41598-023-47343-x)
Supplement: Supplementary file 1 — Supplementary Table 1. [file 41598_2023_47343_MOESM1_ESM.docx]

Supplemental Table 1. Prevalence of coronary microvascular disease stratified by pain to door time.

| Pain to door time (mins) | Direct stenting | Total Patients | CMD | p value |
| --- | --- | --- | --- | --- |
| <120 | no | 33 | 10(30.30%) | 0.028 |
|  | yes | 25 | 1(4.00%) |  |
| ≥120 | no | 79 | 35(44.30%) | <0.001 |
|  | yes | 73 | 11(15.07%) |  |

CMD=coronary microvascular dysfunction
