# Supplementary material for: The impact of primary percutaneous coronary intervention strategies during ST-elevation myocardial infarction on the prevalence of coronary microvascular dysfunction
Source: Sci Rep. 2023 Nov 16;13:20094. doi: 10.1038/s41598-023-47343-x (PMC10654664; doi:10.1038/s41598-023-47343-x)
Supplement: Supplementary file 2 — Supplementary Figure 1. [file 41598_2023_47343_MOESM2_ESM.pptx]

## Slide 1
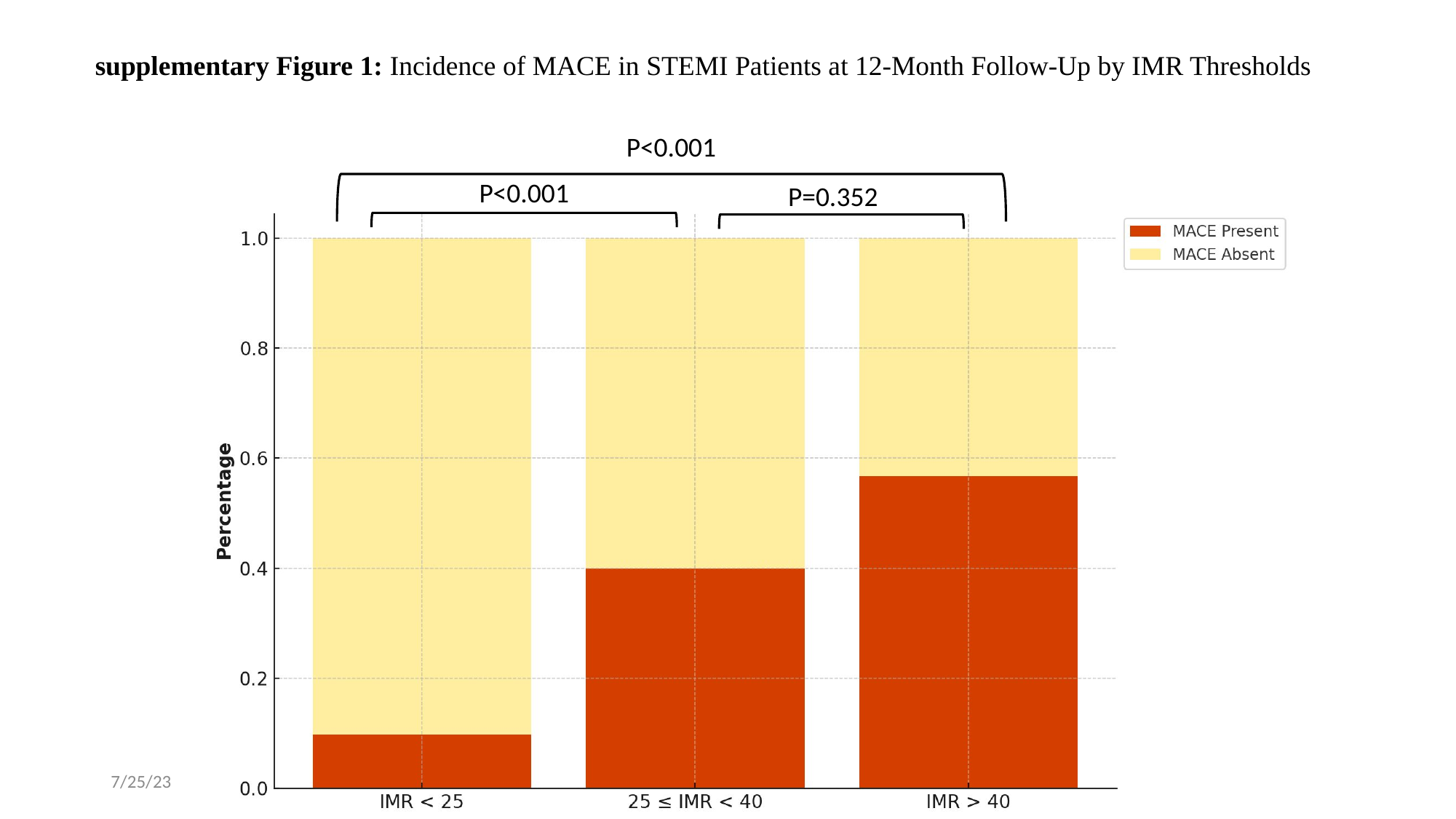

supplementary Figure 1: Incidence of MACE in STEMI Patients at 12-Month Follow-Up by IMR Thresholds
P<0.001
P<0.001
P=0.352
7/25/23
